# Measurement of myocardial blood volume and water exchange using ferumoxytol

**DOI:** 10.1186/1532-429X-17-S1-W11

**Published:** 2015-02-03

**Authors:** Neil Chatterjee, Octavia Bane, Bruce S Spottiswoode, James C Carr, Timothy Carroll

**Affiliations:** 1 Radiology, Northwestern University Feinberg School of Medicine, Chicago, IL, USA; 2 Biomedical Engineering, Northwestern University, Evanston, IL, USA; 3 Radiology, Icahn School of Medicine at Mount Sinai Hospital, New York, NY, USA; 4 Siemens Healthcare, Chicago, IL, USA

## Background

Accurate, quantitative mapping of myocardial blood volume (MBV) could potentially serve as a novel biomarker for cardiovascular disease. However, quantitative measurement of MBV can be technically difficult. Typical gadolinium based contrast agents leak out of the vasculature and are more suited towards measuring extracellular volume than MBV. Even with a completely intravascular contrast agent, water exchange between the intra- and extravascular comparments has been shown to introduce error into MBV measurements. The Hazlewood two comparment model has been used to describe water exchange, and Donahue et al adapted this model to quantify the error water exchange introduces into blood volume measurements. Here we use ferumoxytol, a wholly intravascular iron based contrast agent, to characterize water exchange and quantify MBV in a group of healthy volunteers.

## Methods

5 healthy volunteers were recruited for this study. Each volunteer received multiple small boluses of ferumoxytol (Feraheme). Before and after each bolus, T1 images were acquired using an investigational prototype modified look locker (MOLLI) sequence in a mid-cavity short axis slice (FA 35, TE 1.12, matrix 218x256, 1.4x1.4mm pixel, 8mm slice). All images were acquired on a 1.5T MAGNETOM Aera (Siemens AG, Erlangen, Germany).

For each subject, ROIs were drawn in the myocardium and left ventricular blood pool. Mean T1 values in the ROIs were extracted for each time point and used to calculate the apparent MBV after each bolus (ΔR1 myocardium / ΔR1 blood). The Hazlewood two compartment model was simulated in Matlab to calculate the apparent MBV for any given water exchange rate and true MBV. A least sqaures minimization fitting algorithm was used to determine the water exchange rate and true MBV that best fit the experimental data.

## Results

**Figure 1:**
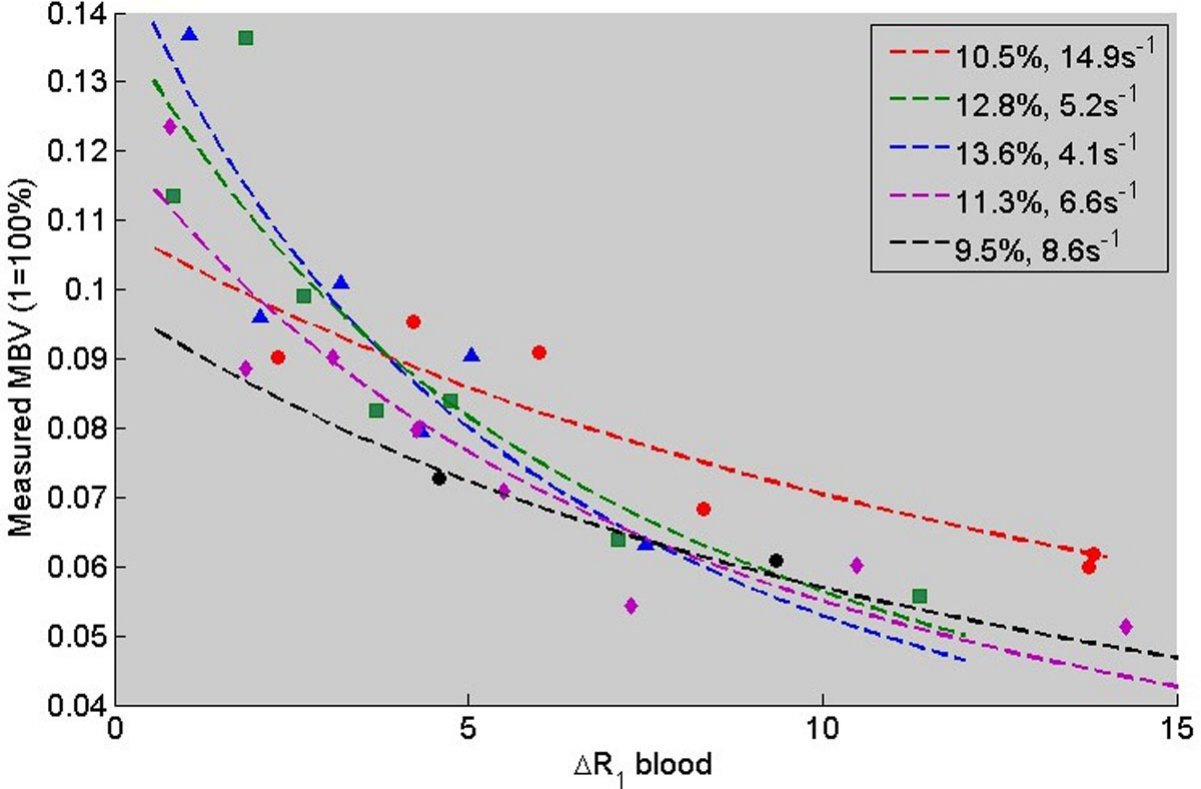
Measured MBV vs blood pool relaxivity in healthy volunteers. Points are experimental data, and lines are simulations of apparent measured MBV vs ΔR1 blood for the best fit true MBV and water exchange frequency.

For all subjects, the fitting algorithm was able to converge on a solution. Fitted experimental data is shown in Figure 1. True MBV was 11.5±1.7% and water exchange frequency was 8.5±4.6s^-1^.

## Conclusions

We were successfully able to measure MBV and water exchange values in human myocardium by measuring T1 changes after serial boluses of an intravascular contrast agent. Our average MBV and water exchange rates of 11.5% and 8.5s-1 were in good accordance with values of 6-12% and 5-9s-1 seen in the literature for animal studies.

From Figure 1, it is clear that water exchange effects can introduce significant errors in MBV measurements. As the change in blood pool relaxivity increases, the apparent MBV (calculated by measured ΔR1 myocardium / ΔR1 blood) increasingly underestimates the true MBV. Without water exchange correction, these errors would manifest as systematic underestimation and scan to scan variability that depends on the contrast dose. However, by correcting for water exchange effects, we were able to recover the true MBV in this cohort of healthy volunteers. More work is needed to investigate how quantitatve MBV correlates with cardiovascular disease.

## Funding

NHLBI (F31 HL117618).

